# Management of Pyogenic Granulomas Following Burn Wounds

**DOI:** 10.29252/wjps.10.3.117

**Published:** 2021-09

**Authors:** Abdolkhalegh Keshavarzi, Mostafa Dahmardehei, Amir Emami, Tayyeb Ghadimi, Behnaz Bouzari

**Affiliations:** 1 Department of Surgery, Shiraz University of Medical Sciences, Shiraz, Iran; 2 Burn Research Center, Iran University of Medical Sciences, Tehran, Iran; 3 Department of Microbiology, Burn and Wound Healing Research Center, Shiraz University of Medical Sciences, Shiraz, Iran; 4 Burn Research Center, Department of Surgery, Iran University of Medical Sciences, Tehran, Iran; 5 Department of Pathology, Firoozgar Hospital, Iran University of Medical Sciences, Tehran, Iran

**Keywords:** Burn, Pyogenic granuloma, Wound

## Abstract

Pyogenic granuloma following burns (PGB) manifest in patients with different clinical and pathological features which is completely different with classic pyogenic granuloma. Up to now, there is no conclusive theory about presents of PG and its accurate etiology. This is a short brief about a 49-yr-old female with, TBSA 1% which PG develop on her burned area after 2 weeks.

## INTRODUCTION

Pyogenic granuloma (PG) or lobular capillary hemangioma which was first named botryomycosis hominis was first described in 1897 by two French surgeons; Antonin Poncet and Dor^1^. This lesion is a vascular tumor that is small, round, and usually bloody red in color. This skin growth occurs on both mucosa and skin and appears as an overgrowth of tissue due to different factors^2^. 

As terminology, the name pyogenic granuloma is misleading as it is neither pyogenic (pus-producing) nor a true granuloma as the cause of hormonal or traumatic and has no association with infection or pus production^3^. Pyogenic granulomas which appear after burn (PGB) is different with those with classic pyogenic granulomas. One of the most important different is related to infectious agent in classic pyogenic granulomas and pyogenic granulomas following burn^4^. In actuality, PGB is divided into two categories according to the large and dramatic clinical changes: proliferating and shriveling stages. Patients who are in the proliferating stage have clinical characteristic consist of various extensive lesions or solitary lesions which may erupt after 1 to 4 wk following burn injury. In these patients, lesions become enlarge and then bleed easily. Clinical characteristics in the shriveling stage seems different substantially. In these victims, lesions are dry, and may form a crust and disappear finally^5^^, ^^6^. 

PGB have three histological features: plasma cell, proliferative vascularization and edematous stromata. One important point which require major attention is related to differentiation of PG with other skin manifestation after burn injuries^7^. A clinician will be able to diagnose these lesions based on their appearance, while for more accurate diagnosis performing a biopsy is recommended. Moreover, clinical and histological characteristic are helpful in this diagnosis, since it required different treatment procedures^8^. 

Herein, we report a case of multiple eruptive PG in a female that developed on burned skin and was treated with Full thickness skin excision.

## CASE PRESENTATION

**Fig. 1:**
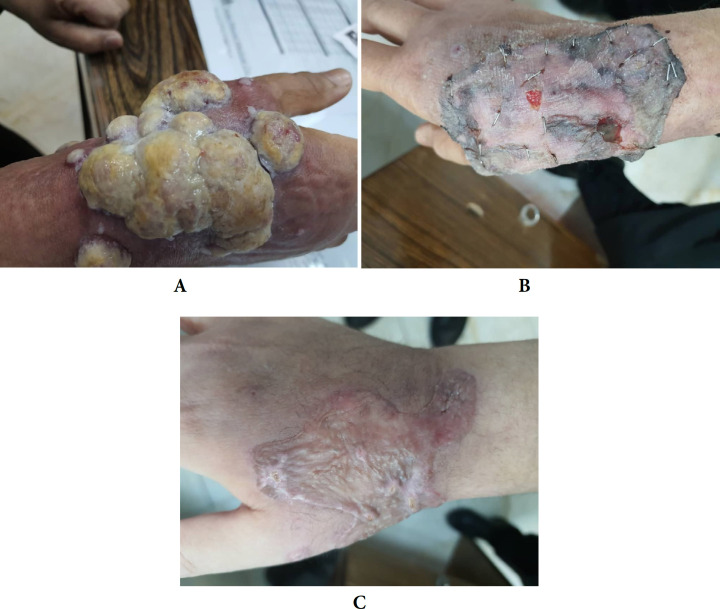
Pyogenic granulomas following burn before (A) and after treatment (B and C)

**Fig. 2:**
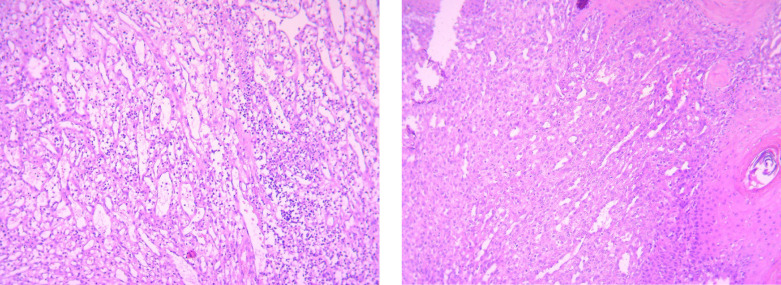
Pyogenic granuloma, lobular pattern of vascular proliferation with inflammation and edema, epidermis at the top with ulceration acanthosis and hyperkeratosis

A 49-yr-old female was hospitalized in Shahid Motahhari Burn Center, Tehran, Iran in 2021, due to deep partial thickness burn with boiling water. Her hand was injured and the total burn surface area was estimated 1%. She was stay at hospital for two days initially. After two weeks, in her referral to outpatient clinic for regular changing dress, some vesicular and pappulomatos was presented in exposed sites which were 5×5×3 cm in size. She was admitted to the hospital for diagnosis and proper care. According to the histopathological examination, the superficial biopsy was shown clinical diagnosis of pyogenic granuloma (Figure1.A). In histologic examination skin tissue show surface ulceration with hyperkeratosis and acanthosis in border of ulcer. The dermis infiltrated by lobular pattern of vascular proliferation and inflammation with areas of edema like granulation tissue formatting. (Figure 2). Based on the patients’ declaration; no pyogenic granuloma development was seen before burn injury. Wound culture during the hospital stay did not show any infectious agent with bacteria or fungi. Full thickness skin excision and debridement was definitive management (Figure 1B). Moreover, skin graft consist of the epidermis and dermis was adhered. Skin graft was taken from right thigh as the donor site. Routine laboratory examinations including (WBC: 4.90, Creatinine: 0.9, Ca:8, K:3.50, Na:131, Hb: 9.70) were all in normal range. During the treatment process no antibiotic was prescribed but according to the patient condition following drugs have been used: Cyclophosphamide, Fluoxetine, isopropanol, prednisolone, and losartan. 

Consent form was obtained for this study and also Shiraz University of Medical Sciences Review Board approved this study by Ethical code: IR.SUMS.MED.REC.1399.589. 

## DISCUSSION

Although the exact etiology is remaining ambiguous for PG and there is no precise reason about the pathogenesis of PG; but various risk factors have been identified for this benign lesion. Trauma is the most frequent risk factor among others^9^. Based on previous evaluations, 50% of patients with PG involve in local trauma ^10^^, ^^11^. However, burn is the most common between trauma. PG, after burn, presents with different clinical features; so it required proper treatment procedures^12^. As it was seen in the current case study, PG was occurred after second degree burn. Interestingly, the majority of patients described with PG in previous reports had experience second degree burn either. Moreover, it was evolved on the burn area during 1-4 wk after burn injury^13^. In the current case, PG was manifested 2 wk following the burn injury. 

In contrast with other reports, wound culture did not show any infection in this case of our study. This is while, *Candida albicans* and *Staphylococcus aureus *was isolated from wound culture^14^. Moreover, *Enterobacter cloacae* was isolated from biopsy tissue and secretion specimen. Viral infection may be one of the infectious agent in PG^15^. 

The causative burn injury was due to hot milk based on the author’s declaration, the justification of PG in those cases may be related to the unknown component in milk^16^. This is while in our case; the burn etiology was hot water. Therefore, it is deducted other factors and mechanisms are responsible for PG manifestation. 

The most important point about PG after burn trauma is conservative treatment. In previous reports full thickness skin excision was choice treatment since no recurrence was seen during 12 months follow-up^17^. Although effective antibiotics and changing dress regularly should not be missed. In the current case, skin graft, excision, and changing dress were performed carefully. Although scars and complications of surgery cannot be avoided.

## CONCLUSION

The eruptive form of PG due to burn is very rare and there are different controversies about the reason and its pathogenicity, so further study, more evaluation and long follow-up are necessary to clarify the ambiguous points in PG after burn trauma. 

## CONFLICT OF INTEREST

None.

## FUNDING

None.
